# Bimodal Glycosyl Donors as an Emerging Approach Towards a General Glycosylation Strategy

**DOI:** 10.1002/chem.202400399

**Published:** 2024-04-17

**Authors:** Matthew E. Warnes, Martin A. Fascione

**Affiliations:** ^1^ Department of Chemistry University of York York YO10 5DD UK

**Keywords:** Stereoselective glycosylation, carbohydrates, glycans, Synthesis, bimodal

## Abstract

Organic synthesis provides an accessible route to preparative scale biological glycans, although schemes to access these complex structures are often complicated by preparation of multiple monosaccharide building blocks. Bimodal glycosyl donors capable of forming both *α‐* and *β*‐anomers selectively, are an emerging tactic to reduce the required number of individual synthetic components in glycan construction. This review discusses examples of bimodal donors in the literature, and how they achieve their stereocontrol for both anomers. Notable examples include a bespoke *O*‐2 benzyl protecting group, a strained glycal for reaction using organometallic catalysis, and a simple perbenzylated donor optimised for stereoselective glycosylation through extensive reaction tuning.

## Graphical abstract

The stereoselective construction of the anomeric bond has constituted one of the greatest challenges in modern organic chemistry. In recent years, examples of “bimodal” glycosyl donors have emerged, demonstrating excellent stereocontrol for both α‐ and β‐anomers. This mini review explores the reactivity and scope of these donors and highlights how they can streamline complex glycan construction.

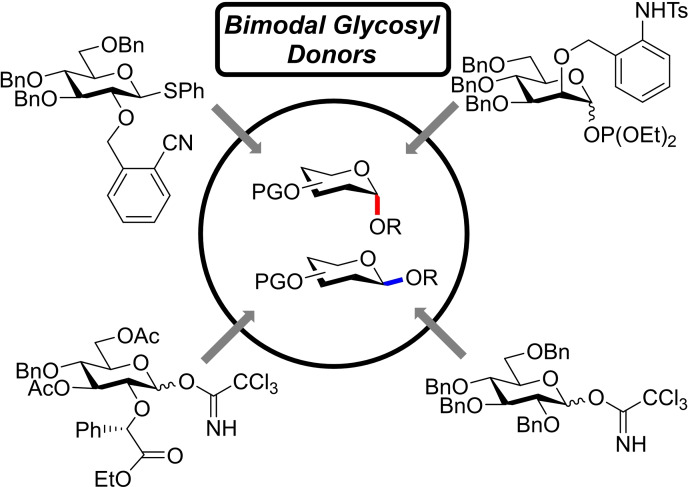

## Introduction

Carbohydrates are among the most ubiquitous biomolecules on earth, with crucial roles in processes such as protein folding,^[1]^ energy storage,^[2]^ cell wall construction,^[3]^ intercellular communication^[4]^ and the immune response.^[5]^ Despite this widespread prevalence and biological significance, their study remains decades behind that of proteins and nucleic acids. This is in part due to their structural heterogeneity, with their *in vivo* construction controlled by a complex array of glycosyltransferase enzymes.^[6]^ Expression and purification of these enzymes offers an *in vitro* approach to obtaining native glycans, but the labour required to prepare extended cascades often limits the utility of these methods.

**Figure 1:**
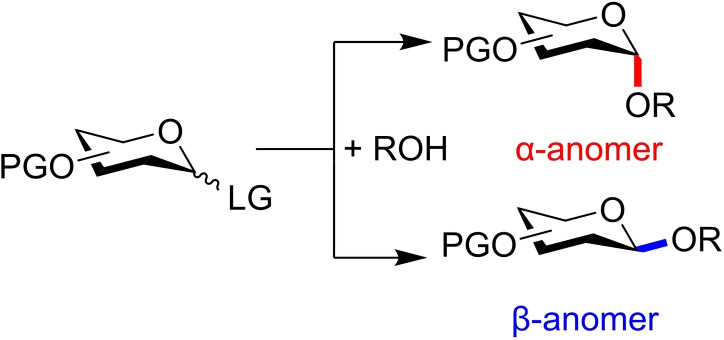
Glycosylations yield two possible anomers, either axial (herein α, as for d‐glucose or other carbohydrates where anomeric configuration is the same as the highest numbered stereocentre) or equatorial (herein β, as for d‐glucose or other carbohydrates where anomeric configuration is the opposite of the highest numbered stereocentre). LG=Leaving group.

As such, chemical synthesis often provides an alternative preparative route to pure glycan samples. Access has however been hampered by the defining chemical step in constructing glycans: the formation of the anomeric bond through glycosylation reactions. Each successive glycosylation results in a new stereocentre, giving either an axial (commonly *α*) or equatorial (commonly *β*) glycosidic linkage (Figure 1), and thus consecutive non‐stereoselective reactions can lead to difficult‐to‐separate isomeric mixtures. Explicit control over this reaction now constitutes a decade‐old problem in organic chemistry, and the requirement for a ‘general glycosylation strategy’ remains a driving force for research in this area.

**Figure 2:**
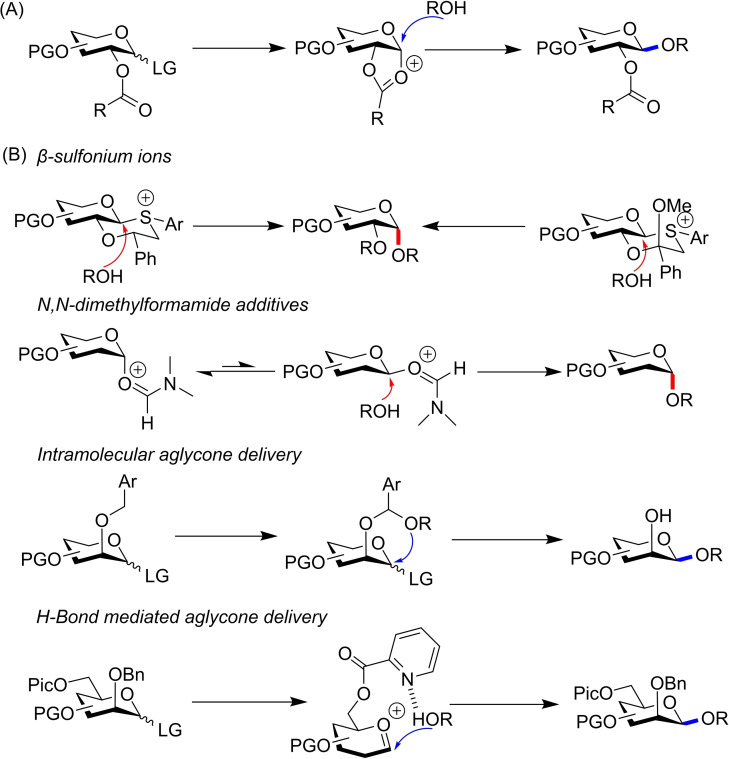
(A) 1,2‐trans‐glycosylations are commonly achieved using O‐2‐acyl groups. (B) Select literature methods for 1,2‐cis glycosylations.

Much of the research in the area of stereoselective glycosylation has focused on separate formation of either 1,2‐*trans‐* or 1,2‐*cis‐*glycosides.^[7]^ The former is routine, with simple O‐2 acyl groups providing complete stereocontrol through neighbouring‐group participation (Figure 2a).^[8]^ The exclusive formation of 1,2‐*cis*‐anomers has proven more challenging,^[9]^ yet highly stereoselective methods now exist for both gluco‐ and manno‐configured donors, including *β*‐sulfonium oxathianes,[^10^, ^11^] *N,N*‐dimethylformamide additives,^[12]^ intramolecular aglycone delivery^[13]^ and H‐bond mediated aglycone delivery (Figure 2b).^[14]^

A less explored approach to controlled glycosylation is the use of powerful ‘bimodal’ glycosyl donors, which are capable of forming either stereoisomer based on the conditions and additives employed. This review focusses on the growing number of bimodal donors, and how they exert their impressive control over stereoselective formation of the anomeric bond.

## Bimodal Glycosyl Donors as a General Glycosylation Strategy

**Figure 3:**
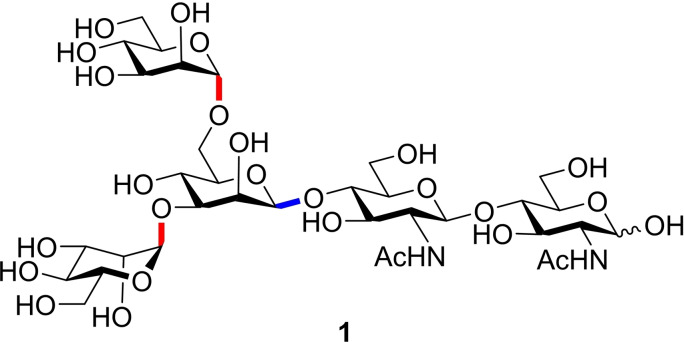
The common core pentasaccharide of eukaryotic N‐glycans.

Stereodivergent glycosylation methods provide clear advantages for glycan synthesis. Using a single donor to construct either anomer can significantly reduce the synthetic effort to prepare all the monosaccharide constituents required in the synthesis of complex oligosaccharides, for example in the *N*‐linked core pentasaccharide glycan fragment **1**,^[15]^ which features *α‐* and *β*‐mannoside linkages (Figure 3). This streamlines construction of glycoconjugates to orthogonal protection strategies, providing the mechanism of bimodality is independent of protecting group derivatisation, and highlights the potential for the development of a commercial library of pre‐bimodal donors, that can simply be protected as required and immediately applied to glycan assembly.

Bimodal donors are therefore a promising approach to greatly simplifying preparation of glycans for biological studies. Current methods to achieve this impressive display of stereocontrol have followed three main approaches; use of bespoke O‐2 protecting groups; transition metal catalysis and careful optimisation of reaction conditions.

## Bimodal Donors Using Bespoke O‐2 Protecting Groups

**Figure 4:**
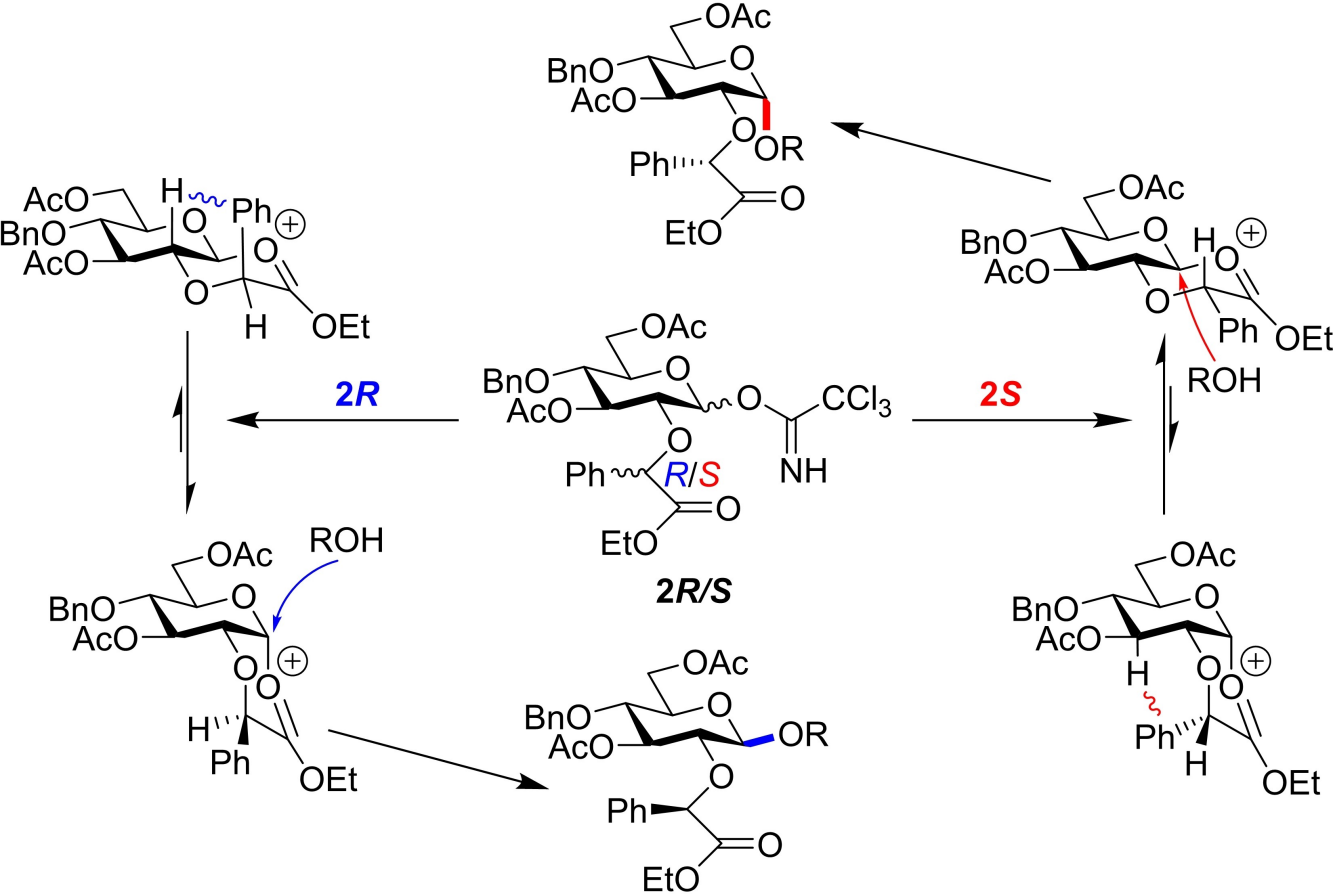
2(R)‐configured auxiliary afford a cis‐decalin like intermediate and β‐glycoside, while the 2(S)‐configured auxiliary proceeds through a trans‐decalin like intermediate that affords the α‐glycoside.

Stereoselective glycosylations have often employed the use of specially designed *O*‐2 groups to infer control over the anomeric bond.^[16]^ Boons and co‐workers reported an early example of bimodal methodology using a trichloroacetimidate donor equipped with an ethyl mandelate auxiliary at the *O*‐2 position **2**.^[17]^ The stereocentre in this functionality controls the major reaction intermediate in the glycosylation, with **2*S*** proposed to form a *trans*‐decalin like structure to prevent phenyl and *H*‐3 steric clash, while **2*R*** forms a *cis*‐decalin like species to avoid unfavourable diaxial interactions (Figure 4). As such, the acceptor nucleophile displaces the auxiliary through S_N_2‐like attack, affording either the α‐1,2‐*cis‐* or β‐1,2‐*trans‐*product depending on the absolute configuration of the *O‐*2 participating group.

**Figure 5:**
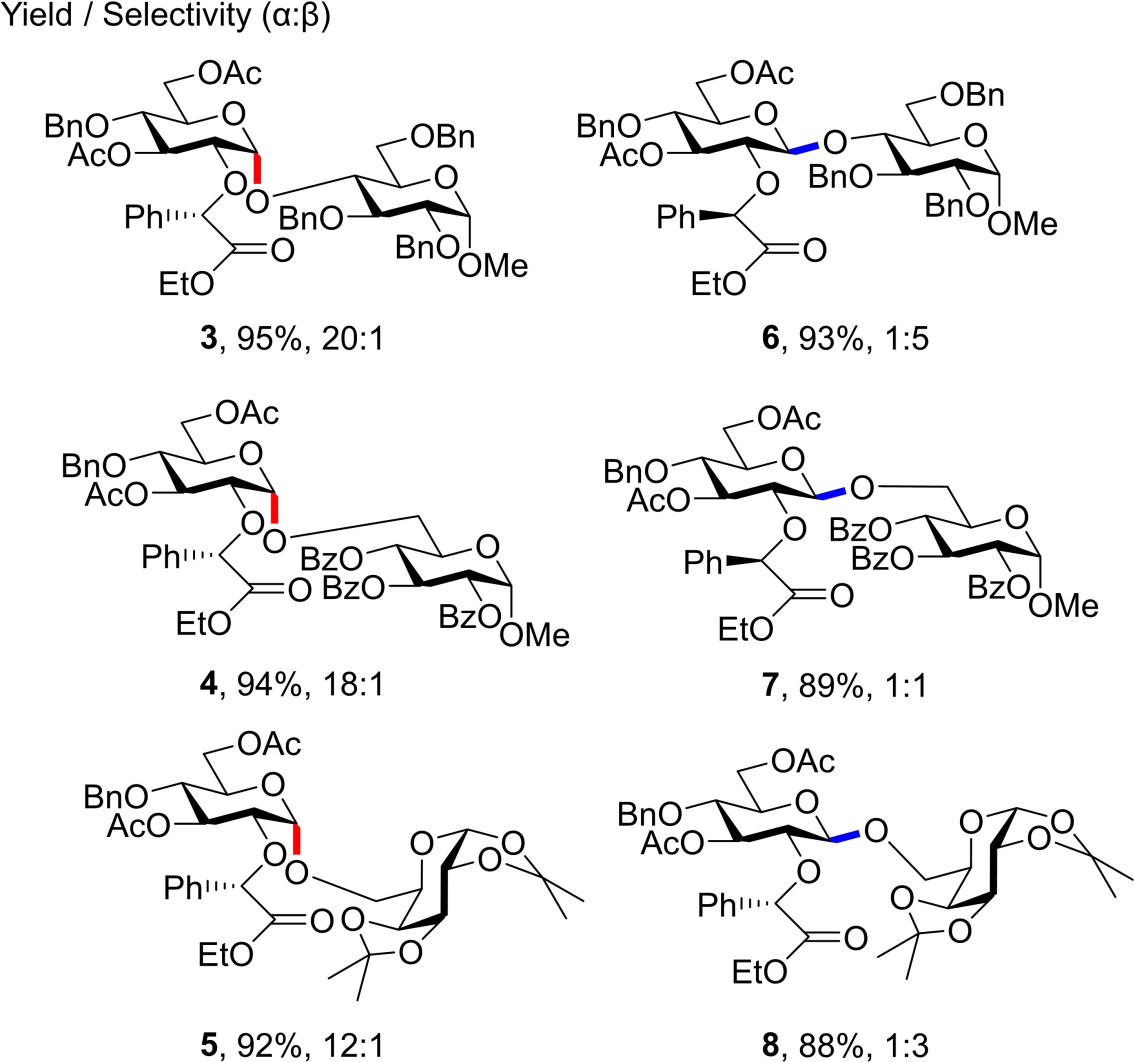
Selected disaccharides formed in Boons and co‐workers study.^[17]^

Glycosylations using auxiliary equipped donor **2** gave excellent yields but varied in selectively (Figure 5). Notably, *α*‐glycosides **3**–**5** formed in significantly greater stereoselectivity compared to the corresponding *β*‐glycosides **6**–**8**, with unwanted anomer formation attributed to S_N_1‐like attack on oxocarbenium intermediates. Although *β*‐selectivity was demonstrated in this work, later‐generation auxiliaries focused solely on 1,2‐*cis*‐glycosylations and so no more bimodal donors with this methodology have been reported.

**Figure 6:**
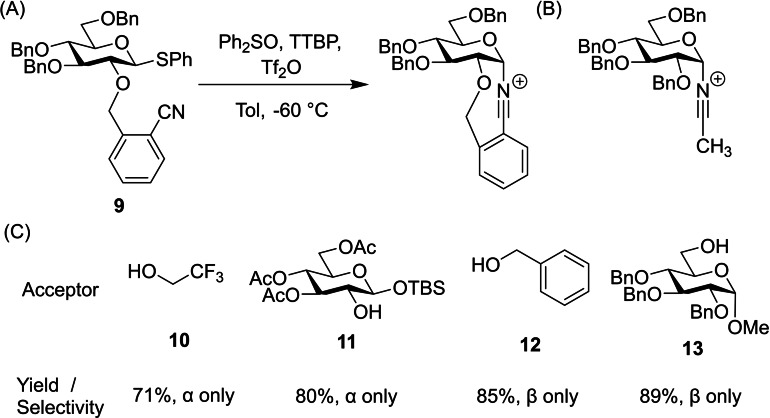
(A) The 2‐cyanobenzyl ether protecting group forms an α‐nitrilium ion resembling speciation in nitrile solvent. (B) Electron poor acceptors show α‐selectivity but electron rich acceptors show β‐selectivity.

Another example of an *O*‐2 protecting group facilitating donor bimodality was reported by Hoang and Liu, using a 2‐*O*‐cyanobenzyl ether protected donor **9**.^[18]^ The authors installed a cyano moiety on the *O*‐2 benzyl protecting group, aiming to exploit the *β*‐selectivity often observed in nitrile solvent (Figure 6a and 6b).^[19]^ However, an acceptor screening gave a serendipitous discovery: complete reversal of the intended stereoselectivity when using electron poor acceptors. When electron rich alcohols were employed, the donor reacted with complete *β*‐selectivity, but moving to acceptors such as 2,2,2‐trifluoroethanol **10** instead lead to *α*‐glycoside formation (Figure 6c).

**Figure 7:**
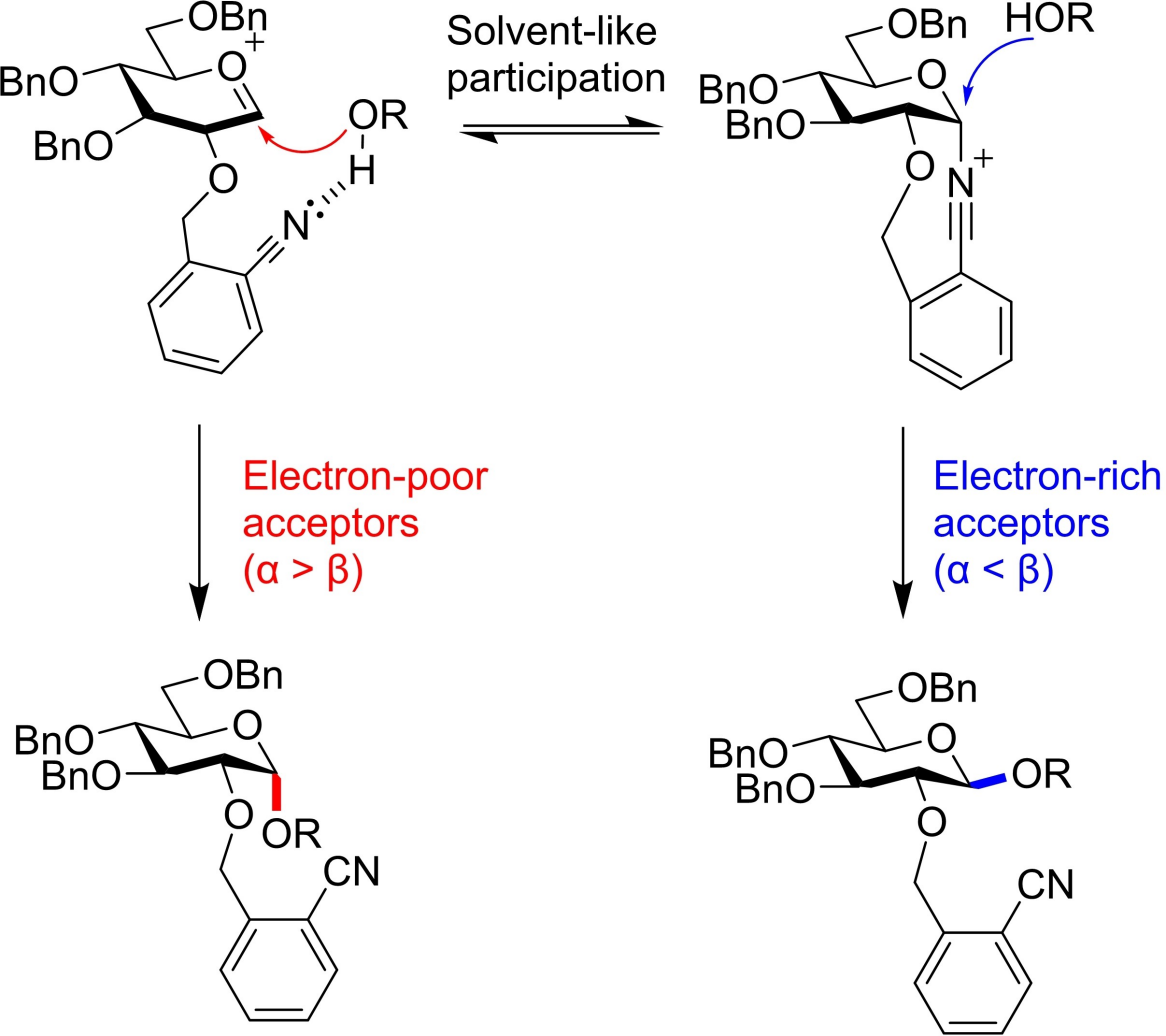
Stronger nucleophiles are able to displace the α‐nitrilium ion, whereas weaker nucleophiles are guided to the α‐face by the dissociated 2‐O‐cyanobenzyl group.

Mechanistic studies using low temperature NMR and H‐bond disrupting additives reinforced a tentative mechanistic hypothesis. For electron rich acceptors such as **12** and **13**, anomeric bond formation proceeds through simple S_N_2 displacement of the α‐nitrilium ion to give the *β‐O*‐glycoside. Conversely, electron poor acceptors like **10** and **11** are too weakly nucleophilic to achieve this displacement, and so instead are guided to the α‐face by an intermolecular hydrogen bond with the dissociated nitrile moiety (Figure 7).

Despite this system's impressive stereoselectivities, the synthetic scope is severely limited by the acceptor structure determining the reaction outcome. The potential for an *O*‐2 benzyl auxiliary as a bimodal donor was evident however, and so future efforts explored similar motifs.

**Figure 8:**
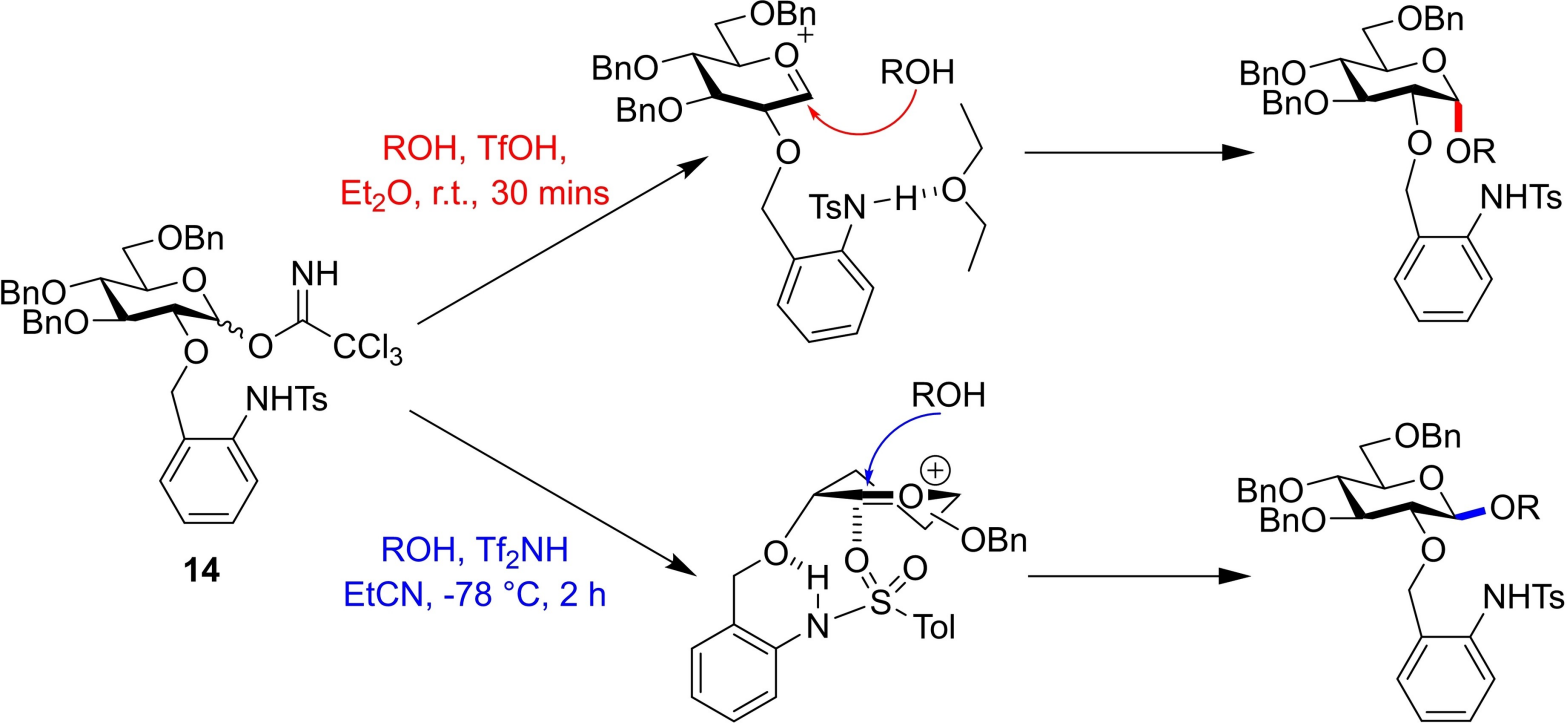
Under α‐promoting conditions, ether solvent disturbs the hydrogen bonding and normal pyranose α‐preference is observed. In EtCN, an intramolecular H‐bond leads to the α‐face being blocked, and therefore gives the β‐anomer.

Ito and co‐workers later developed donor **14**, instead protected with the 2‐*O*‐(*ortho*‐tosylamido)benzyl ether (TAB) group (Figure 8).^[20]^ In this system, stereoselectivities are governed by the hydrogen bonds formed by the tosylamido functionality. In propionitrile solvent, a proposed internal H‐bond between the amido proton and benzyl oxygen forms, allowing *α*‐coordination of the tosyl oxygen. As this protects the *α*‐face, the incoming nucleophile approaches to afford the *β*‐glycoside. When the hydrogen bonding is disturbed in ethereal solvents, the TAB ether no longer influences the reaction, and the general *α*‐selectivity directed by the anomeric effect for glycosylations of *O*‐2 benzyl donors is realised.

**Figure 9:**
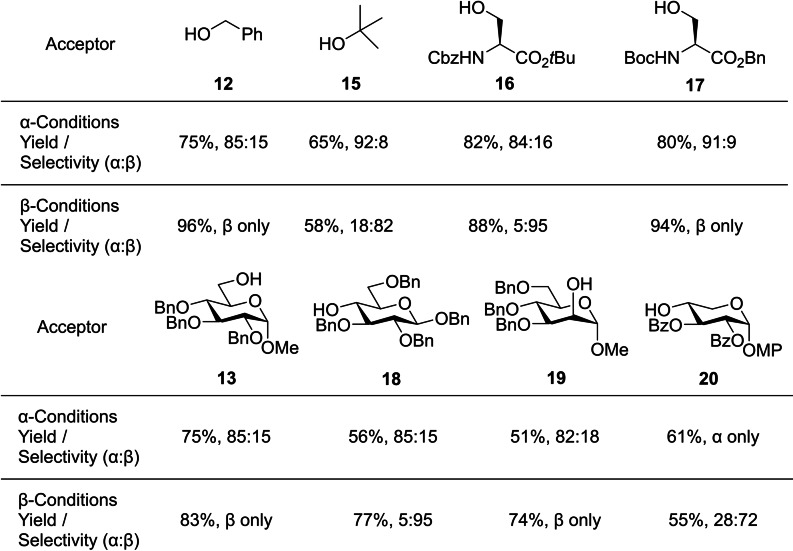
Selected results from acceptor screening of donor **14** with various alcohols. MP=4‐methoxyphenyl.

Glycosylations using donor **14** displayed good yields and generally high selectivities. However, the non‐exclusive method for generating α‐anomers was apparent in the lower selectivities compared to the β‐ conditions. Notably, many of the β‐glycosylations proceeded with complete stereocontrol, whereas this was only observed under α‐selective conditions for xylose acceptor **20** (Figure 9). Selective deprotection of the *O*‐2‐TAB protecting group was also demonstrated, as well as successful cleavage under standard debenzylation conditions.

**Figure 10:**
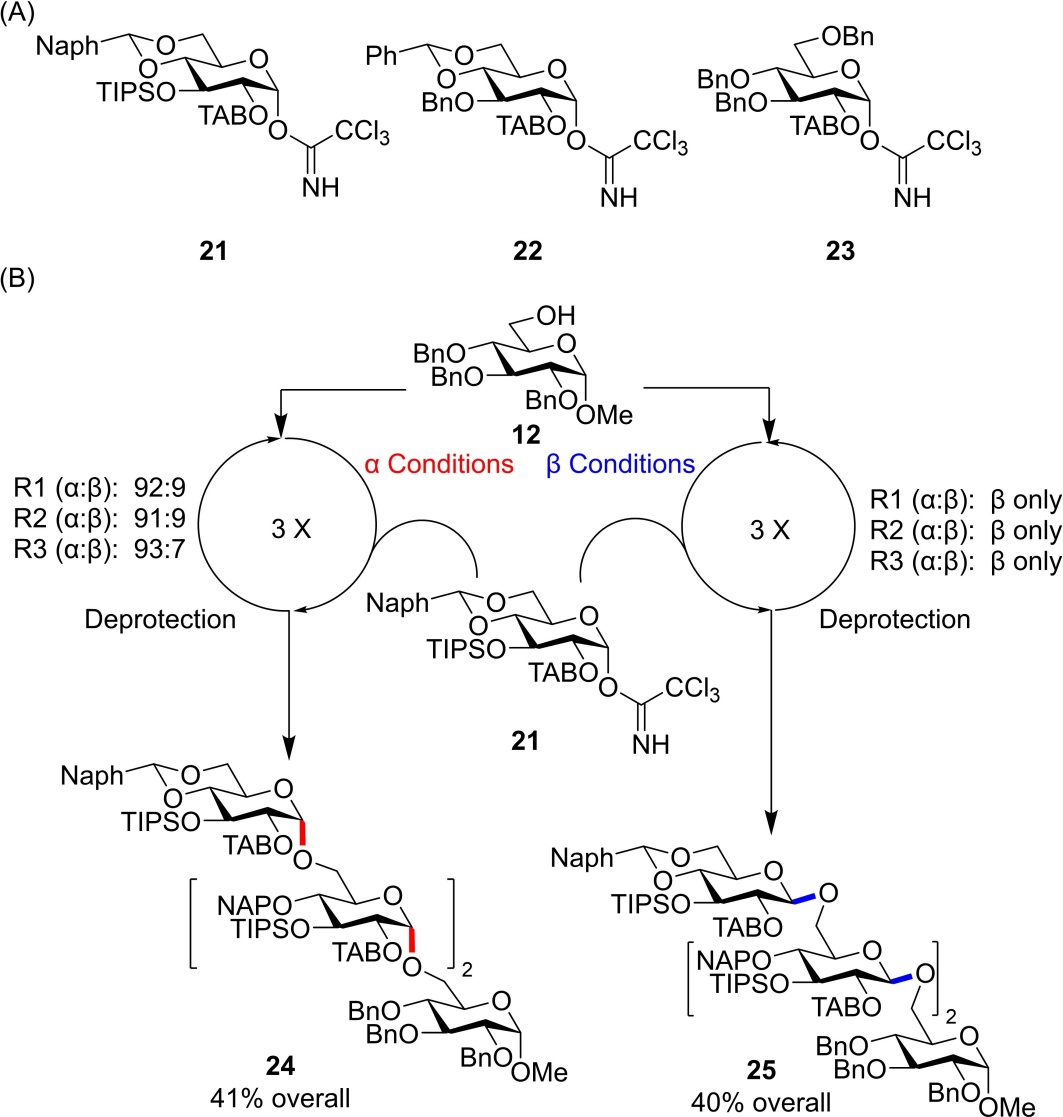
(A) Donors used for glycan construction. (B) Three consecutive (R1, R2, R3) glycosylations/deprotections using donor **21** affords the α,α,α‐ or β,β,β‐tetramaltosides. α Conditions: TfOH, Et_2_O (0.004 M), r.t.; β Conditions: Tf_2_NH, EtCN (0.1 M) −78 °C; Deprotection Conditions: BH_3_⋅THF, TMSOTf, DCM.

The same authors later published results demonstrating the advantage of a bimodal approach to complex oligosaccharide construction.^[21]^ Using just three imidate donors **21**, **22** and **23** with different protecting group patterns (Figure 10a), both linear and branched oligosaccharides were synthesised with high stereocontrol over each glycosidic bond. The same donor **21** was used to construct the tetramaltosides **24** and **25** with either all *α*‐ or all *β‐(1→6)‐*linkages (Figure 10b).

**Figure 11:**
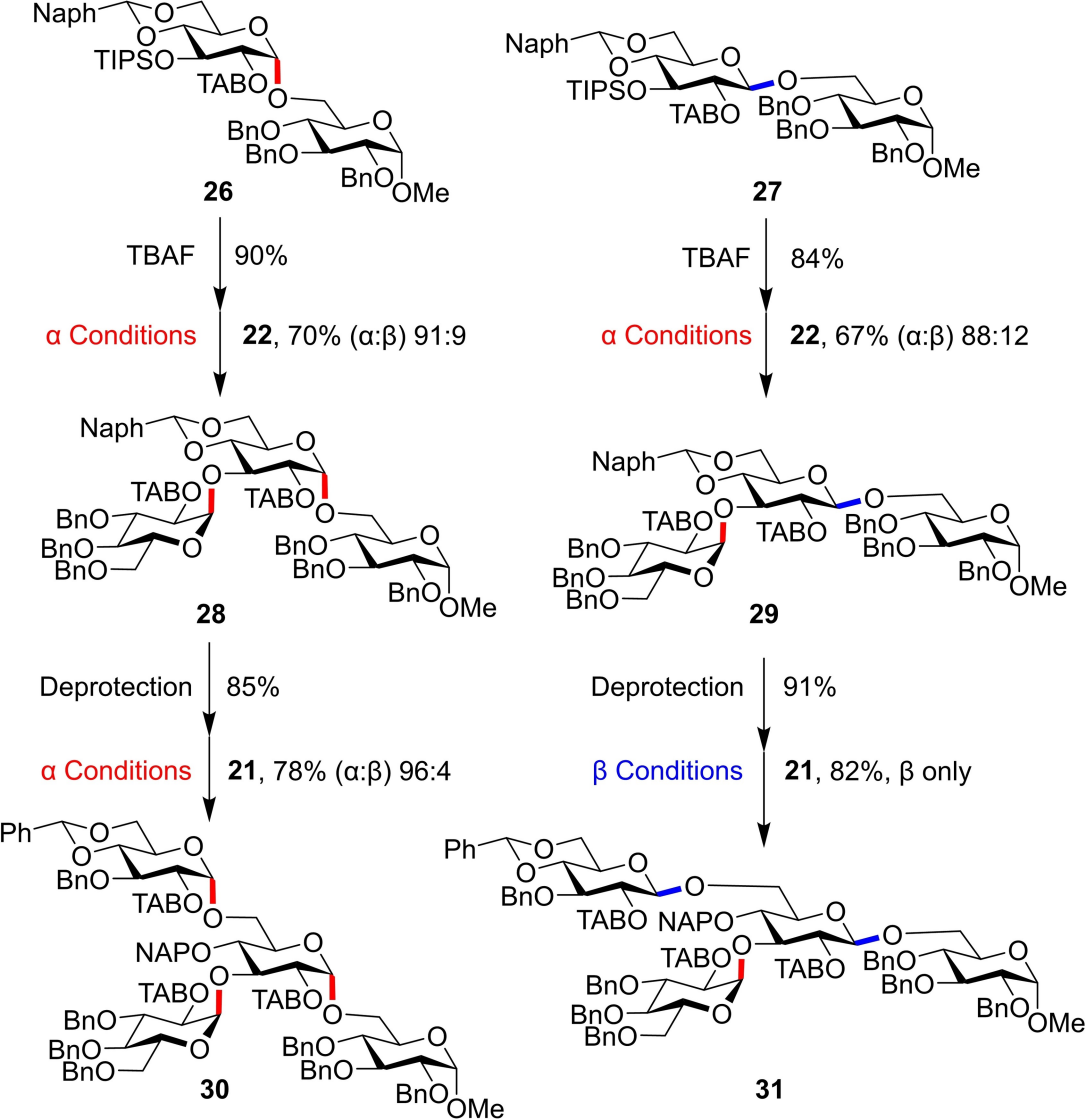
Construction of branched maltosides through two successive glycosylations. α Conditions: TfOH, Et_2_O (0.004 M), r.t.; β Conditions: Tf_2_NH, EtCN (0.1 M) −78 °C; Deprotection Conditions: BH_3_⋅THF, TMSOTf, DCM.

The bimodal approach was also demonstrated for branched maltosides **30** and **31** (Figure 11) starting from the *α‐* and *β*‐disaccharides **26** and **27** constructed previously. Selective *O*‐3 deprotection and subsequent *α*‐glycosylation allowed addition of **23**, followed by 4,6‐*O*‐benzylidene ring opening and bimodal glycosylations using **22**.

**Figure 12:**
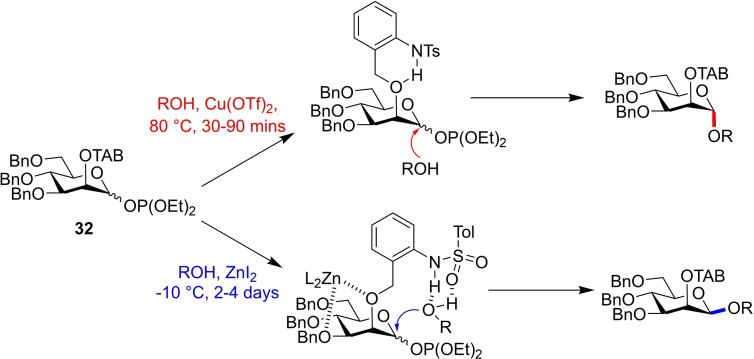
α‐selectivity occurs due to stereoelectronic effects of mannose donors, as the TAB ether does not participate. ZnI_2_ breaks the O‐2/tosylamido H‐bond, allowing guidance of the incumbent nucleophile to the β‐face.

The TAB ether protecting group has also shown promise in the development of a bimodal mannosyl donor.^[22]^ Ito and co‐workers again applied their novel protecting group, this time to a mannose phosphite donor **32**. In this example, stereocontrol is instead induced by a Lewis acid additive, either Cu(OTf)_2_ for *α*‐selectivity or ZnI_2_ for *β*‐selectivity. NMR experiments again indicated the presence of an intramolecular hydrogen bond in the TAB ether, leading to a similar mechanism to be proposed. In the presence of Cu(OTf)_2_, the TAB ether's internal hydrogen bond is undisturbed, and so again results in *α*‐selectivity due to the anomeric effect.^[23]^ Alternatively, ZnI_2_ is able to coordinate to *O*‐2, liberating the tosylamido proton which subsequently guides the incoming nucleophile to the *β*‐face (Figure 12).

**Figure 13:**
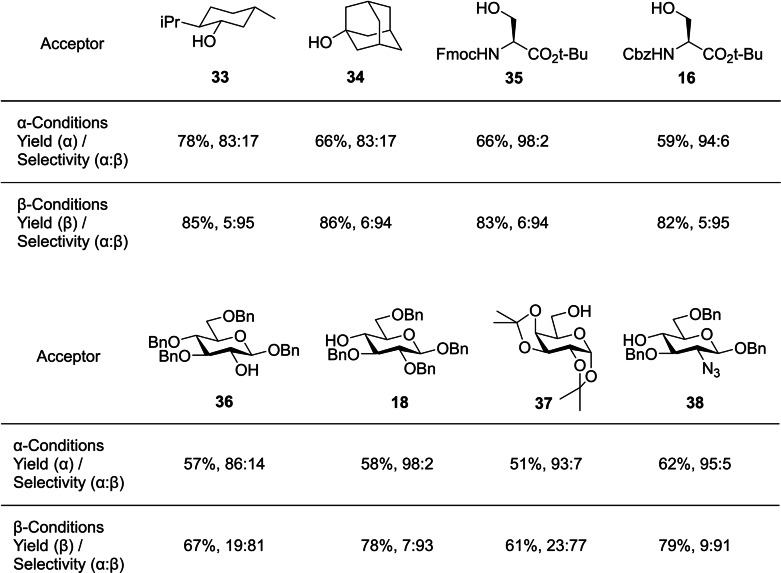
Selected glycosylations of phosphite donor **32** with various acceptors.

TAB ether equipped mannosylations showed good selectivities, with a wide range of acceptors tested including protected amino acids (Figure 13), though the extended reaction time required for *β*‐selectivity limits the synthetic utility of this donor for constructing high mannose type glycans.

Clearly, O‐2 protecting groups are an excellent approach to realising stereodivergence in glycosyl donors, whether it be through further optimisation of TAB ether based approaches, or the development of new auxiliaries. However, other approaches to bimodality have avoided the effort of installing specific protecting groups regioselectively, and so offer a more potentially more economical route to preparative scale glycan synthesis.

## Glycals as Transition Metal Catalysed Bimodal Donors

Glycosylation chemistry is one of the many fields that have benefitted from recent advances in organometallic catalysis.^[24]^ Notably, glycals have been shown to be excellent substrates for metal‐controlled reactions due to their 1,2‐unsaturated systems that present a handle for the action of d‐block catalysts.^[25]^ Of course, many examples of stereoselective glycosylations using these systems exist,[^26^, ^27^, ^28^] but methods that allow for construction of both *α*‐ and *β*‐adducts are still rare.

**Figure 14:**
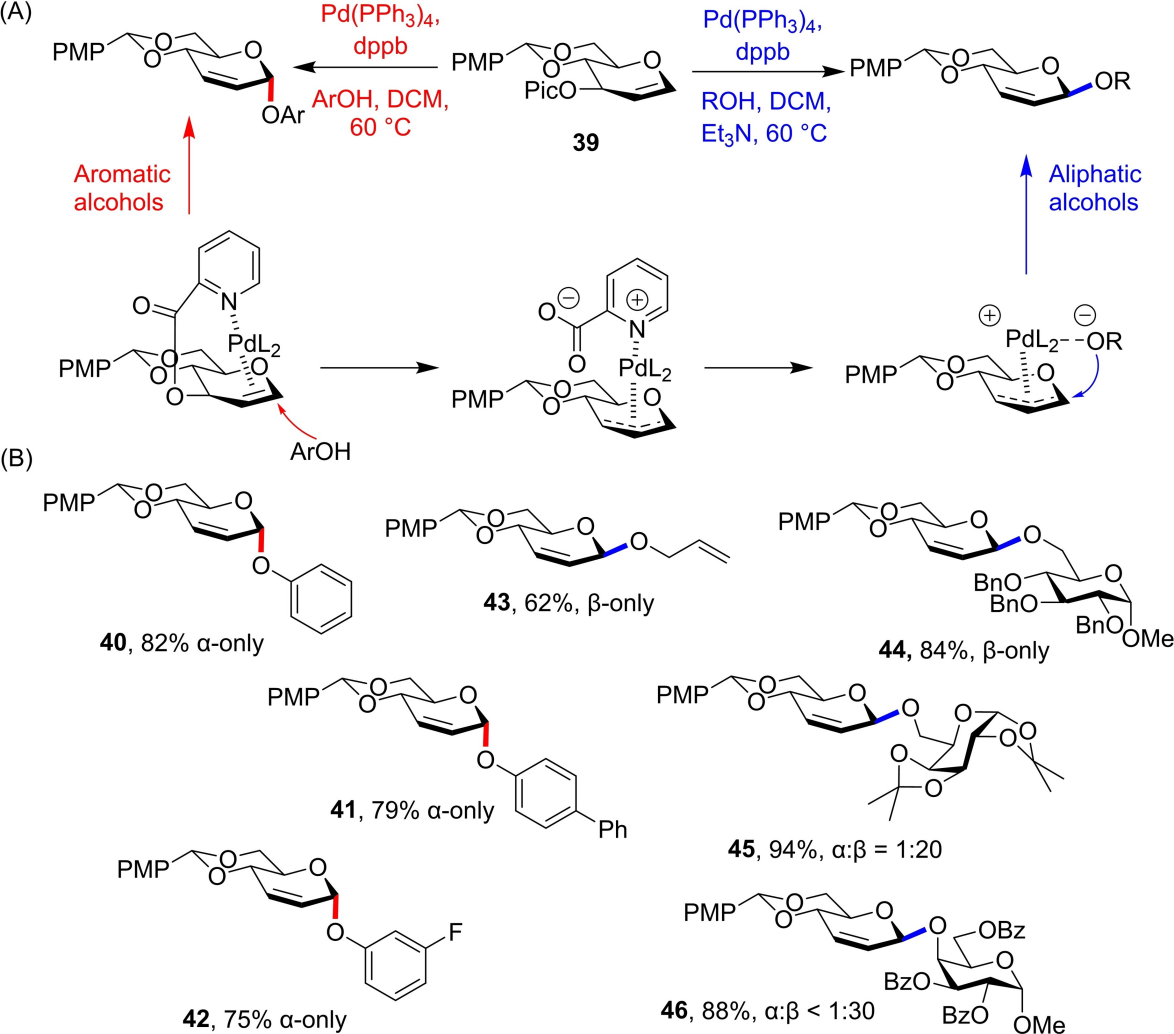
(A) Aromatic alcohols give α‐glycosides through an outer sphere mechanism, while aliphatic alcohols give β‐glycosides via coordination to the Pd centre. (B) Selected glycosides formed using donor **39**.

Liu and co‐workers have reported a bimodal methodology based on 3‐*O*‐picoloyl protected glucal **39**.^[29]^ The authors hypothesised a Pd metal centre would coordinate to the 1,2‐unsaturated π‐system and picoloyl moeity from the top face of the donor (Figure 14a). Thus, as in Trost‐Tsuji‐type reactions,^[30]^ harder nucleophiles would attack through an inner‐sphere mechanism affording the *β*‐glycoside, while weaker nucleophiles could approach through an outer sphere mechanism that gives the *α*‐product. Indeed, glycosylations using phenol acceptors gave example glycosides **40**–**42** in excellent *α*‐selectivity, while use of aliphatic and glycosyl acceptors gave the *β*‐products **43**–**46** in impressive stereopurity with triethylamine as an additive (Figure 14b). Glycal Donor **39** therefore shows stereodivergence dictated by acceptor electronic properties, similar to 2‐cyanobenzyl ether donor **9**.

**Figure 15:**
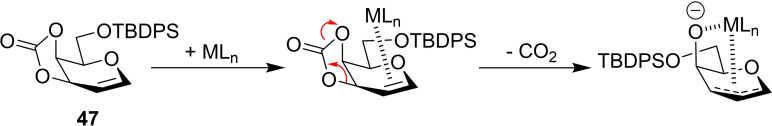
Coordination of the unsaturated system in galactal donor **47** results in CO_2_ release and allyl ligation to the catalyst.

Yao and co‐workers later presented two similar methodologies for bimodal glycosylations with glycals, now using 3,4‐*O*‐carbonate galactal donor **47** (Figure 15). The *cis*‐carbonate provides driving force for organometallic activation of an allyl system through a strained C3‐*O* bond and subsequent release of CO_2_.

**Figure 16:**
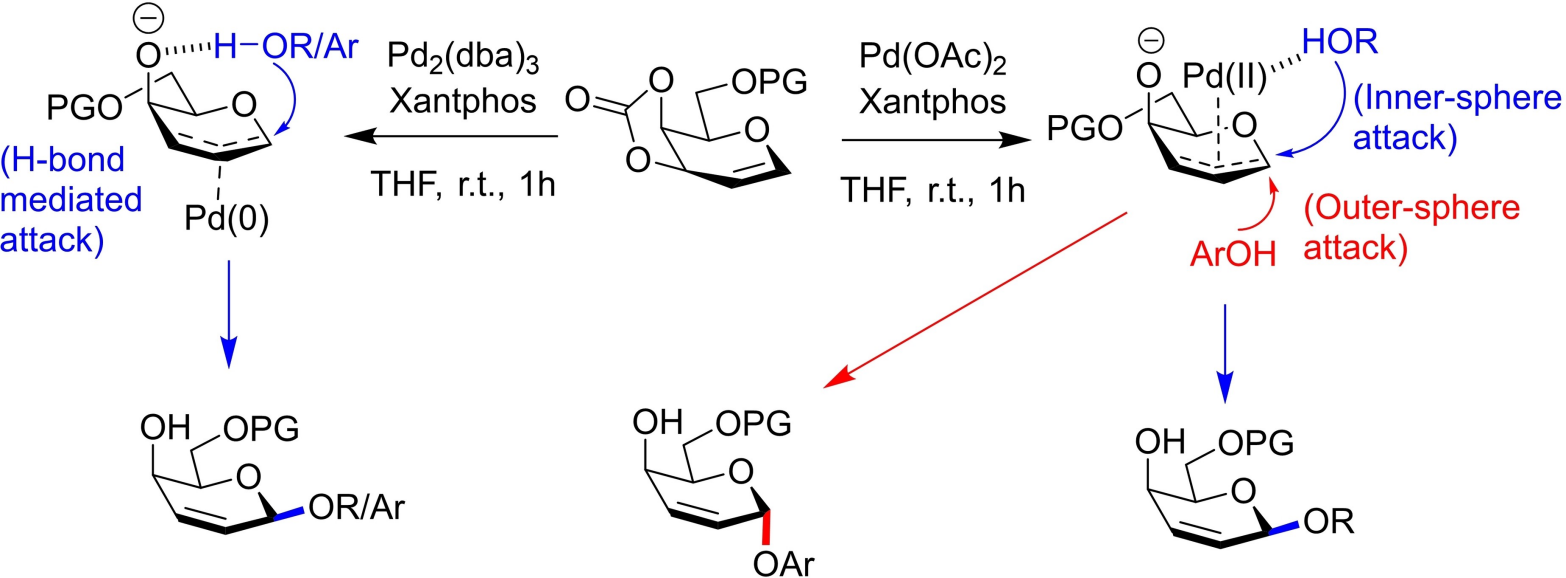
Explanation for observed stereoselectivities using galactal donor **47**. Pd(0) catalysts coordinate to the bottom face of the allyl system, affording only β‐glycosides. Pd(II) can afford α‐glycosides through an outer sphere pathway, or the β‐glycosides through an inner sphere mechanism.

**Figure 17:**
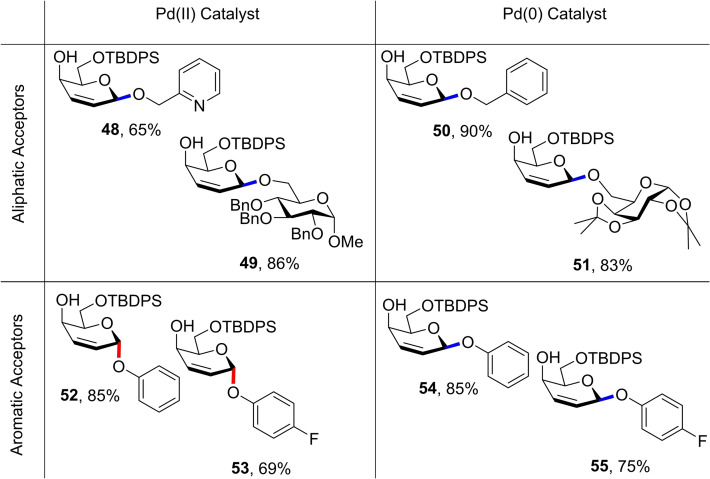
Selected results from glycosylations using Pd(II)/Pd(0) catalysts and aliphatic/aromatic alcohol acceptors. All selectvities >50 : 1 for the intended anomer.

Their first report utilising donor **47** inspected the influence of Pd(II) and Pd(0) catalytic species on glycosylation outcome.^[31]^ Initial reaction screenings using Pd(II) and hard aliphatic alcohol acceptors showed complete *β*‐selectivity due to inner‐sphere guidance by the Pd(II) centre. The authors reasoned that using a softer alcohol acceptor such as phenol would instead lead to an outer‐sphere pathway culminating in *α*‐attack by the nucleophile (Figure 16 and Figure 17) as previously observed with donor **39**. Acceptor screenings confirmed this, with exclusive *α*‐selectivity being observed for a variety of aromatic alcohols with good yields.

It was further reasoned that using a Pd(0) catalyst would alternatively lead to catalyst coordination to the bottom face preventing steric clash between the axial O‐4 and the larger Pd(0) species. As such, all alcohols would attack from the *β*‐face, aided by a directive hydrogen bond from the *O*‐4‐alkoxide. Indeed, reactions using Pd_2_(dba)_3_ furnished the glycals as *β*‐2,3‐unsaturated glycosides with a variety of both aliphatic and aromatic acceptors (Figure 16 and Figure 17).

**Figure 18:**
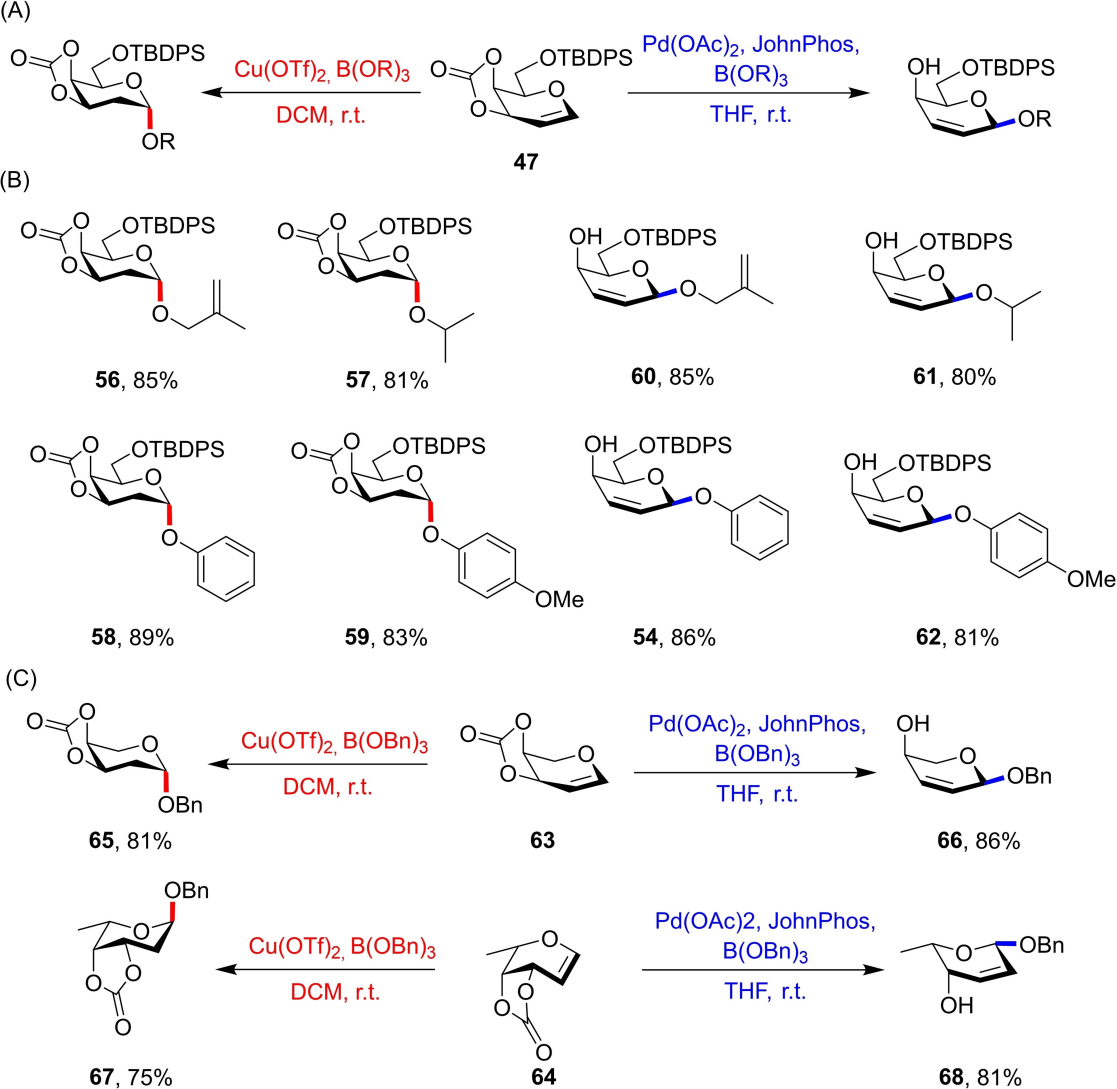
(A) Optimised conditions for α‐ and β‐selective reactions. (B) Select results from glycosylations using both conditions. (C) An arabinal and fucal donor showed equally impressive stereocontrol. All selectivities >50 : 1 for the intended anomer.

3,4‐*O*‐Carbonate galactal **47** acted as a bimodal glycosyl donor but only for phenol acceptors, severely limiting its synthetic utility to specific aglycans. This was later addressed by the same authors where they reported the use of boronate ester acceptors, allowing reaction with aliphatic acceptors while maintaining complete control over anomeric stereochemistry.^[32]^ However, while access to the *β*‐glycosides still proceeded through a Pd catalysed cycle, *α*‐selectivity was induced using a Cu(OTf)_2_ Lewis acid catalyst, and now instead formed 2‐deoxy‐3,4‐*O*‐carbonate‐*α*‐glycosides (Figure 18a).

**Figure 19:**
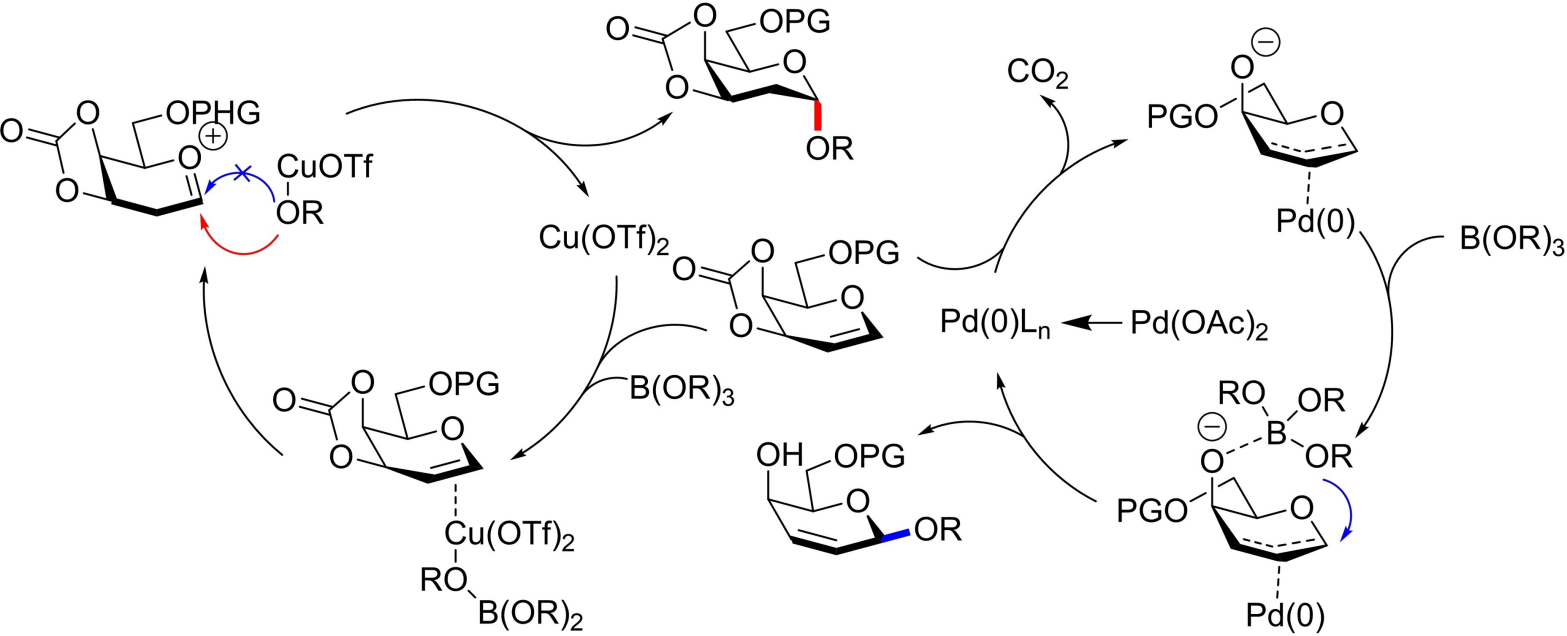
Proposed mechanism for Cu(OTf)_2_ and Pd(OAc)_2_ boronate glycosylations.

A tentative *β*‐glycosylation mechanism suggested the previous activation of the carbonate via an *in situ* generated Pd(0) species coordinated to the bottom face. The alkoxide then guides the boronate acceptor to attack at the anomeric position with *β*‐selectivity. For 2‐deoxy‐*α*‐glycoside synthesis, approach of the Cu(OTf)_2_ polarises the glycal unsaturated system, leading to conversion to the oxocarbenium. Approach of the Cu‐coordinated alcohol acceptor proceeds from the lower face due to steric considerations, giving the *α*‐stereoisomer (Figure 19).

For both anomers, yields and stereoselectivities were excellent (Figure 18b), with extension to glycal configurational isomers including an arabinose **63** and fucose **64** donor affording benzyl glycosides **65**–**68** (Figure 18c).

With this advance, 3,4‐*O*‐carbonate glycals now acted as bimodal donors with both aliphatic and aromatic alcohols, although no glycosylations using glycosyl acceptors were demonstrated. Additionally, the requirement for a glycal donor limits the post‐glycosylation functionalisation available, making this method more appropriate for constructing pharmaceutical frameworks than native glycans.

Although their synthetic versatility is apparent, the use of rare earth organometallic catalysts also constitutes a more complicated reaction system. As such, many studies have avoided the use of these compounds, instead opting to extensively tune standard glycosylation conditions. This methodology has been applied to a vast library of glycosyl donors and gives a simplified approach to achieving bimodal stereochemical control.

## Conditions‐Tuning Approach to Bimodal Donors

A significant body of literature in glycosylation chemistry has focused on the effects of simple conditions changes to control anomeric selectivity.^[33]^ Parameters such as solvent,^[34]^ concentration,^[35]^ promoter^[36]^ and temperature^[37]^ can be varied to give often dramatic effect on the resulting stereochemical mixture. As such, achieving bimodal reactivity may be as simple as finding optimal conditions for both *α*‐ or *β*‐glycoside formation.

Additionally, the advent of high‐throughput methodologies presents an efficient method for rapidly screening glycosylation conditions,^[38]^ and may even provide access to data‐driven approaches for predicting selectivities on novel donors.^[39]^ This ultimately paves the way towards general glycosylation strategies where donor bimodality may be accessible for almost any scaffold, potentially simplifying oligosaccharide synthesis.

**Figure 20:**
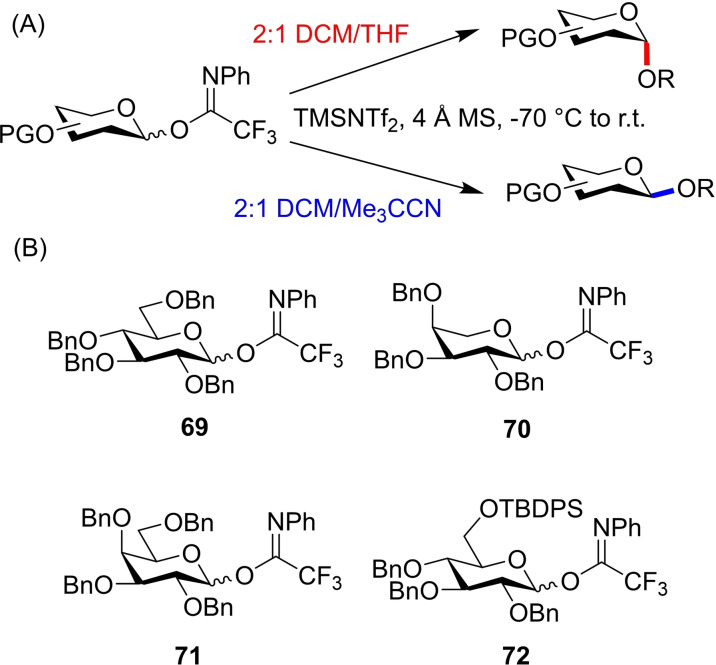
(A) Conditions for α‐ and β‐selective glycosylations in the study by Yu and co‐workers.^[40]^ (B) Imidate donors used in the study.

Complete selectivity without the addition of any auxiliary or exotic catalyst has been demonstrated elegantly by Yu and co‐workers where the synthesis of ‘amycellulose’, an alternating *α*/*β*‐oligoglucoside, is described.^[40]^ Using simple *N*‐phenyltrifluoroacetimidate donor **69** the authors were able to demonstrate complete stereoselectivity for both anomers, where only solvent choice was varied. 2 : 1 DCM/THF solvent mixture afforded the *α*‐anomer selectively, while nitrile containing 2 : 1 DCM/Me_3_CCN furnished the *β*‐glycoside (Figure 20a). All other conditions, including temperature, concentrations and promoters were constant, demonstrating the profound influence of solvent on stereochemical outcome.

**Figure 21:**
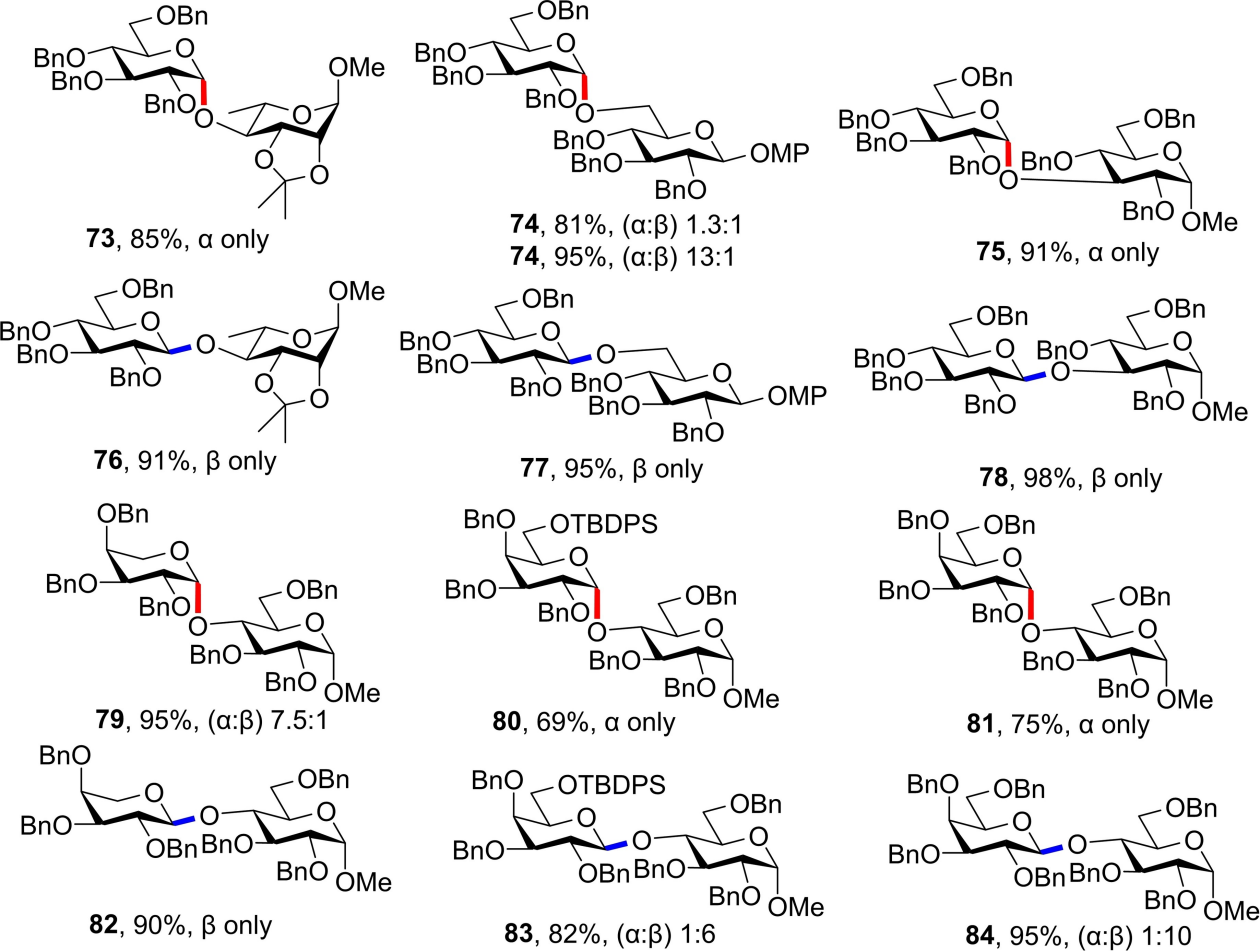
Select glycosides synthesised in the study by Yu and co‐workers.^[40]^

The reaction scope for imidate donor **69** was promising, showing excellent yields and selectivities with a range of glycosyl acceptors affording disaccharides **73**–**78**. Notably, where *α*‐selectivites were poorer for glycoside **74**, a *N,N*‐dimethylformamide additive improved the selectivity, demonstrating the conditions‐tuning based approach (Figure 21). The same set of conditions were also applied to donors **70**–**72** (Figure 20b) affording glycosides **79**–**84**, with only a small reduction in stereocontrol, but still excellent yields (Figure 21). Whether minor tuning of glycosylation conditions could regain complete stereocontrol in these scaffolds has not yet been explored.

**Figure 22:**
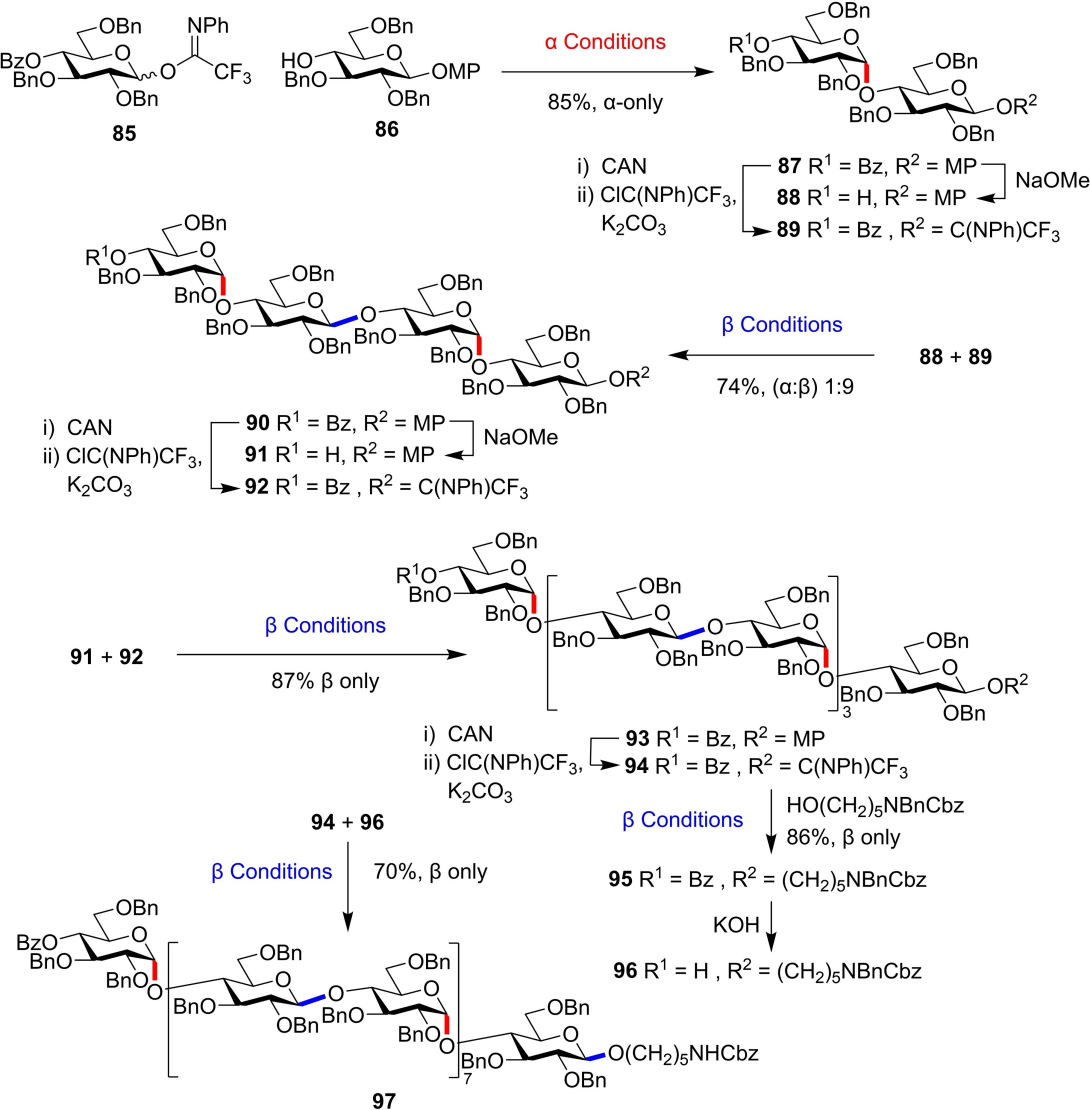
Synthesis of amycellulose 16mer from donor **85** and acceptor **86**. α Conditions: TMSNTf_2_, 2 : 1 DCM/THF, 4 Å MS, −70 °C to r.t.; β Conditions: TMSNTf_2_, 2 : 1 DCM/Me_3_CCN, 4 Å MS, −70 °C to r.t. MP=4‐methoxyphenyl.

This methodology was subsequently applied to the synthesis of the *α*/*β*‐alternating oligosaccharide ‘amycellulose’. Starting from acceptor **86**, the unnatural glycan was constructed using a convergent [2^*n*^+2^*n*^] glycosylation strategy with 4‐OBz protected donor **85** (Figure 22). Owing to the excellent anomeric selectivities, a 16mer of amycellulose **97** was accessible using simple deprotection and glycosylation steps, allowing preparative isolation and subsequent conformational study of this unique glycan.

**Figure 23:**
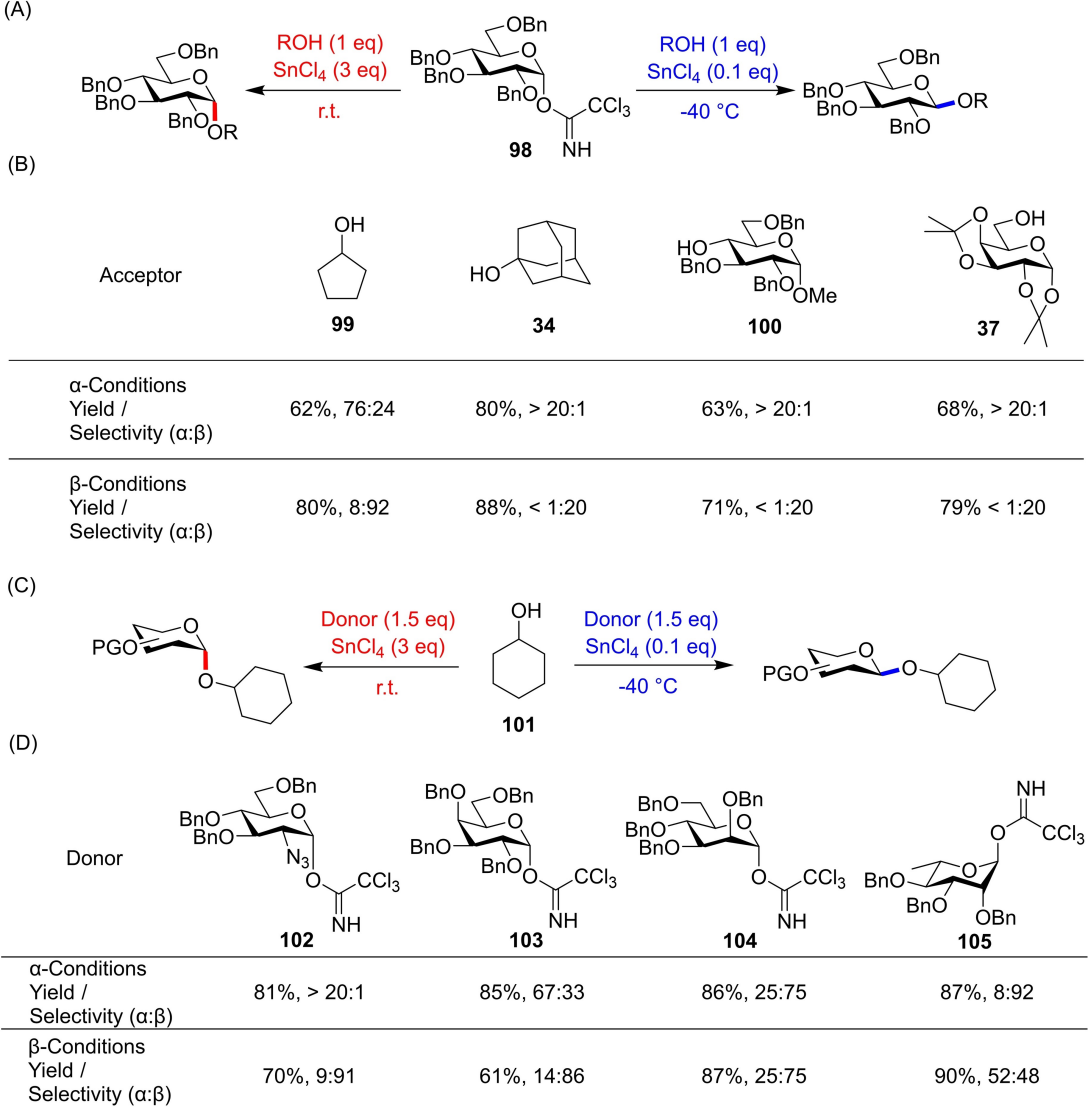
(A) Conditions for α‐ and β‐selective reactions. (B) Selected acceptors and associated yields/selectivities. (C) Conditions for different donor scaffolds. (D) Results of different donor scaffolds with cyclohexanol acceptor **101**.

Another simple method for bimodal glycosylations was reported by Ding and co‐workers, now using a similar perbenzylated trichloroacetimidate **98** (Figure 23a).^[41]^ The stereocontrol was exerted through addition of SnCl_4_, with catalytic amounts and low temperatures giving the *β*‐anomer, whereas excess Lewis acid afforded the *α*‐anomer after extended reaction times at room temperature. An acceptor screening using benzylated α‐glucose donor **98** gave generally excellent selectivities and yields (Figure 23b). However, changing to other α‐donor scaffolds **102**–**105** saw a large reduction in stereocontrol for both *α*‐ and *β*‐glycosides (Figure 23c, d) It has not yet been reported whether re‐optimisation of conditions for each donor alleviates this issue.

**Figure 24:**
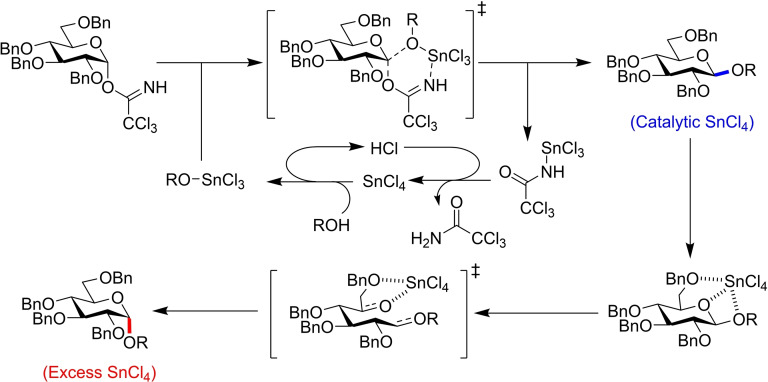
Catalytic mechanism for β‐glycoside formation using donor **98**, then mutarotation mechanism for α‐glycoside formation.

A controlled model experiment and accompanying DFT calculations provided mechanistic insight. Under catalytic conditions, *α*‐trichloroacetimidate donors are activated by an acceptor‐tin adduct that promotes S_N_2 displacement (Figure 24), with the catalyst regenerated through a proton transfer pathway. This affords the *β*‐glycoside, which can then be converted to its *α*‐counterpart by mutarotation. At higher temperatures and with excess of SnCl_4_, coordination of the Lewis acid leads to endocyclic cleavage, affording formation of the more thermodynamically stable *α*‐glycoside.^[42]^ These simple condition changes allow for stereodivergent behaviour in this glucose donor under facile conditions, and so application to other scaffolds and protecting group strategies would prove an excellent glycosylation approach.

**Figure 25:**
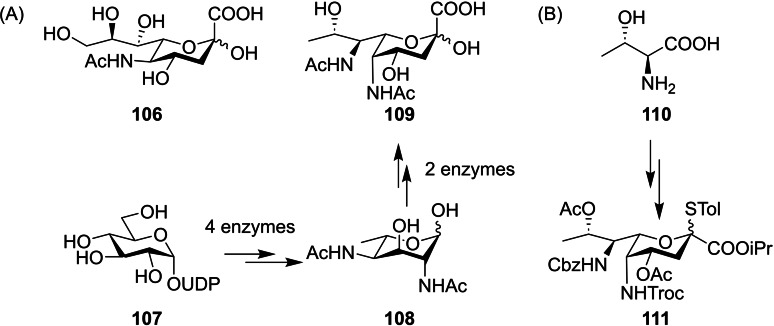
(A) Neu5Ac **106** is ubiquitous in mammalian cell surface glycoconjugates, Pse5Ac7Ac **109** is a bacterial analogue biosynthesised from UDP‐GlcNAc **107** through intermediate L‐Alt‐2,4‐DiNAc **108**. (B) Literature de‐novo synthesis of Pse5Ac7Ac thioglycoside **111** used for glycosylation.

Of course, interest in chemical glycosylation is not limited to pyranose scaffolds. The nonulosonic acids are a family of 9‐carbon ketoacid sugars that are prominent in cell surface glycoconjugates,^[43]^ particularly Neu5Ac **106** which is expressed as the glycobiological human ‘sign‐of‐self’^[2]^ (Figure 25a). Analogous to Neu5Ac is the bacterial sialic acid‐ pseudaminic acid (Pse5Ac7Ac) **109**, which is of interest in immune evasion.^[44]^ Access to glycans containing Pse5Ac7Ac **109** is limited by both its lengthy preparation^[45]^ and lack of characterised glycosyltransferase enzymes,^[46]^ so effective chemical methods for preparation of its native glycans are highly desired. Li and co‐workers described a method for bimodal chemical pseudaminylation^[47]^ based on a synthetic thioglycoside **111** prepared through a route they reported previously (Figure 25b).^[48]^

**Figure 26:**
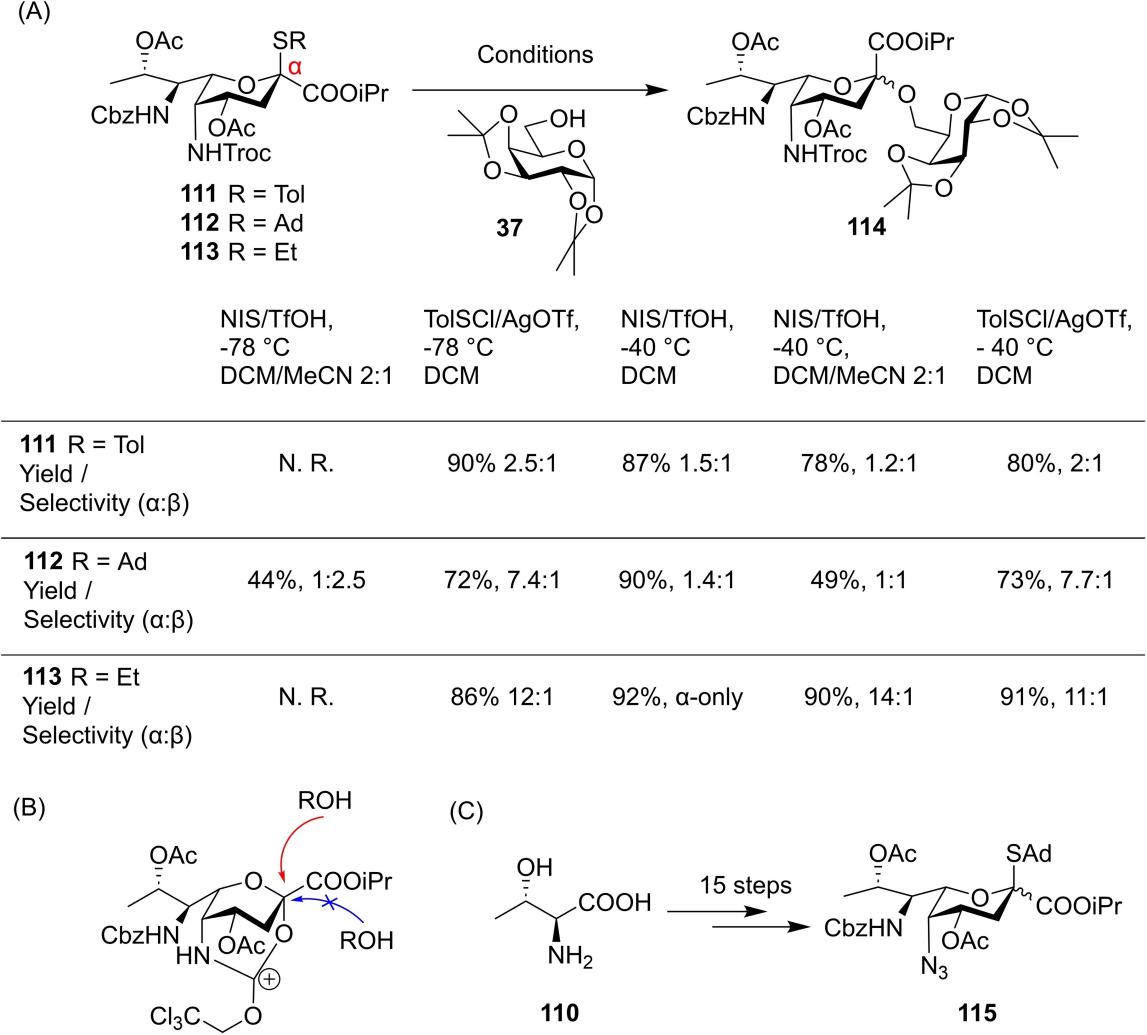
(A) Initial optimisation for 5 *N*‐Troc donors **111**–**113** showed preference for α‐glycosides. (B) Proposed rationale for α‐selectivity. (C) Synthesis of 5 *N*‐azido derivative **115** achieved through adapted route previously reported.

Initial optimisations began by varying the thiol aglycone between STol **111**, SAd **112** and SEt **113** while changing glycosylation conditions (Figure 26a). The less reactive **111** and **113** failed to activate at −78 °C with *N*‐iodosuccinimide and trifluoroacetic acid, but otherwise results displayed a clear *α*‐anomer preference for all three donors and all solvent/promoter conditions. The authors hypothesised that remote participation from the 5 *N*‐Troc moiety was responsible for this α‐selectivity (Figure 26b), and so a 5 *N*‐azido **115** was prepared through a modified synthesis (Figure 26c).

**Figure 27:**
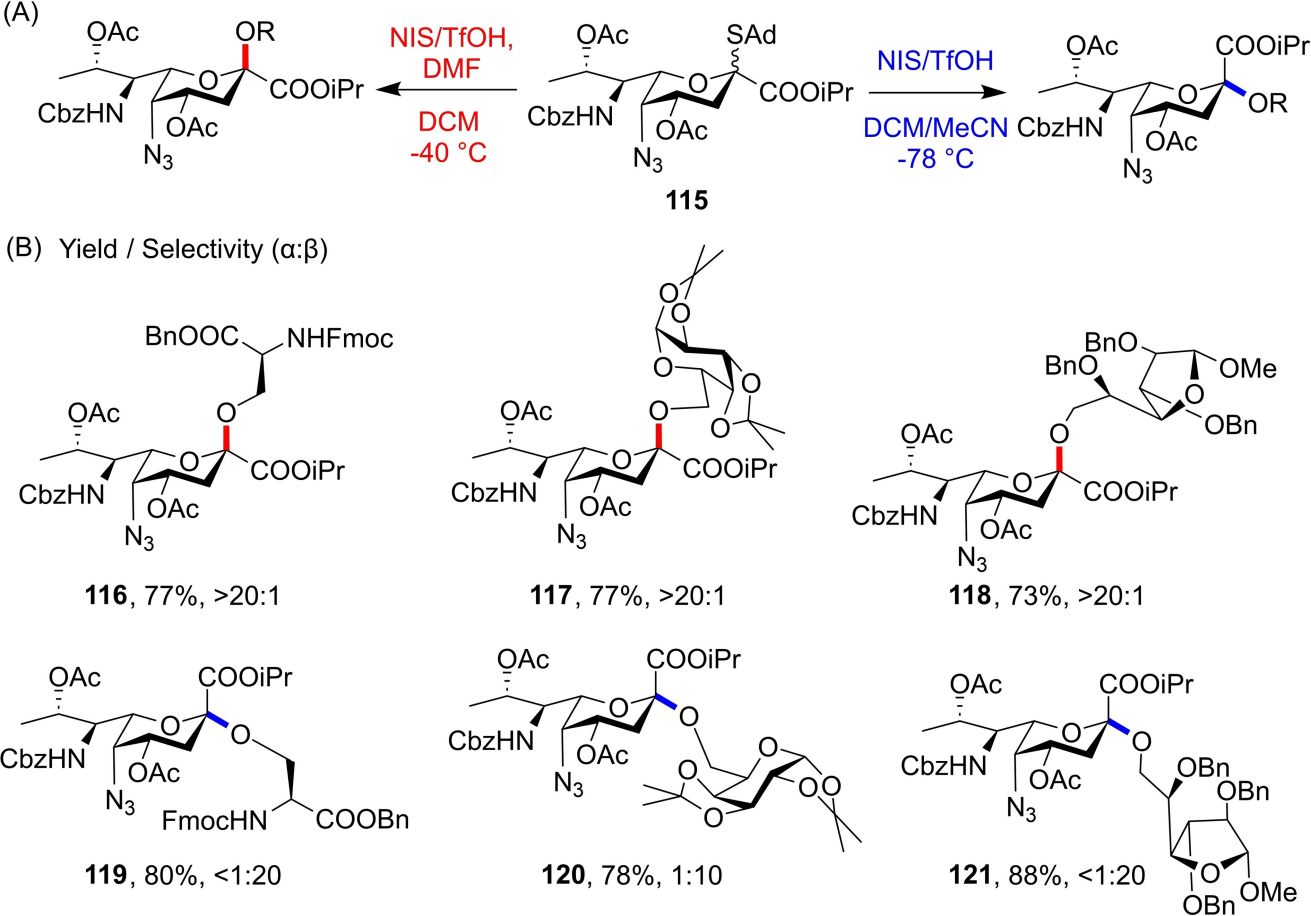
(A) Optimised conditions for stereoselective formation of α‐ or β‐anomers using donor **115**. (B) Selected glycosides synthesised using the optimised conditions.

The nonparticipating azido group in **115** allowed for excellent *β*‐selectivity with a variety of acceptors in a DCM/MeCN solvent mixture, and glycosylations with *N,N*‐dimethylformamide as an additive allowed both 5 *N*‐Troc **112** and 5 *N*‐azido **115** to afford the *α*‐anomers (Figure 27a), giving access to glycosides such as **116**–**121** (Figure 27b). Therefore, in this example azido nonulosonate **115** functions as a bimodal donor, ensuring that access to these exotic glycans is limited only by supply of pseudaminyl donors and not non‐stereoselective glycosylations. However, requirement of a 5 *N*‐azido functionality restricts the possible synthetic routes to access the donor, particularly chemoenzymatic routes through the late stage biosynthetic intermediate L‐Alt‐2,4‐DiNAc **108** which much work has focused on.[^49^, ^50^, ^51^] As such, development of a similar Pse5Ac7Ac donor that shows greater *N*‐derivatisation tolerance is desirable.

## Conclusions

These examples make clear the promise bimodal glycosyl donors offer to expediting multistep stereoselective glycan synthesis. Whether future advances are made through bespoke designed C2 auxiliaries, specially armed donors or extensive reaction tuning, all these approaches offer a potential route to a long sought‐after general glycosylation strategy.

However, there is also space for innovation in the design of new bimodal glycosylation approaches, particularly in the development of leaving groups with the potential to coordinate the approach of the incoming acceptor nucleophile to either face.^[52]^ A bimodal glycosylation strategy also has yet to be applied to automated glycan assembly,^[53]^ where the greatest synthetic utility compared to traditional glycosylation approaches may be realised. There are also few examples of bimodal donors outside of the pyranose and glycal scaffolds, and little consideration of donor performance in synthesising *N*‐ and *C‐*glycosides. This is particularly apparent for studies of nucleosides and their analogues, where no methods exist for stereodivergent *N*‐glycosylation of pentose sugars.

Despite this potential, stereodivergent donors remain an underexplored field that could further streamline synthetic routes to biologically relevant glycans on a preparative scale.

## Conflict of interests

The authors declare no conflict of interest.

1

## Biographical Information

*Matthew Warnes is a current M.Chem. student at the University of York (UK), where he will begin Ph.D. studies in the Fascione group in October 2024 on synthetic carbohydrate chemistry. Matthew's current research is aimed at discerning the structural influences on the selectivity and reactivity of glycosyl donors, with the goal of developing synthetically robust building blocks for stereoselective glycosylations*.

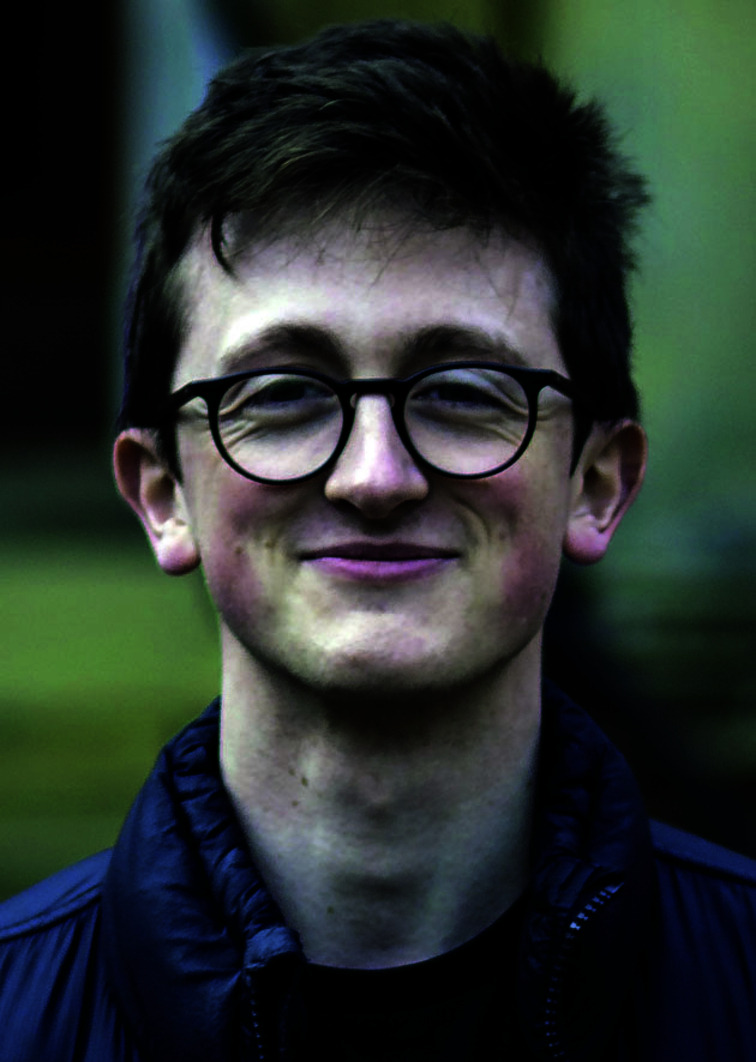

## Biographical Information

*Dr. Martin Fascione leads an interdisciplinary glycoscience research group within the Department of Chemistry at the University of York, UK. Martin received his Ph.D. from the University of Leeds in 2009 under the supervision of Prof. W. Bruce Turnbull on the stereoselective synthesis of 1,2‐cis‐glycosides. Following a postdoctoral period in Leeds, he was then awarded a Marie Curie Fellowship to study the mechanisms of carbohydrate processing enzymes with Prof. Steve Withers, FRS, at the University of British Columbia in Vancouver and Prof. Gideon Davies, FRS, FMedSci, at the University of York, UK. In August 2014 he took up a lectureship in in York. His research interests include the chemical glycobiology of nonulosonic/sialic acids, synthetic and enzymatic carbohydrate chemistry, and the bioconjugation of proteins*.

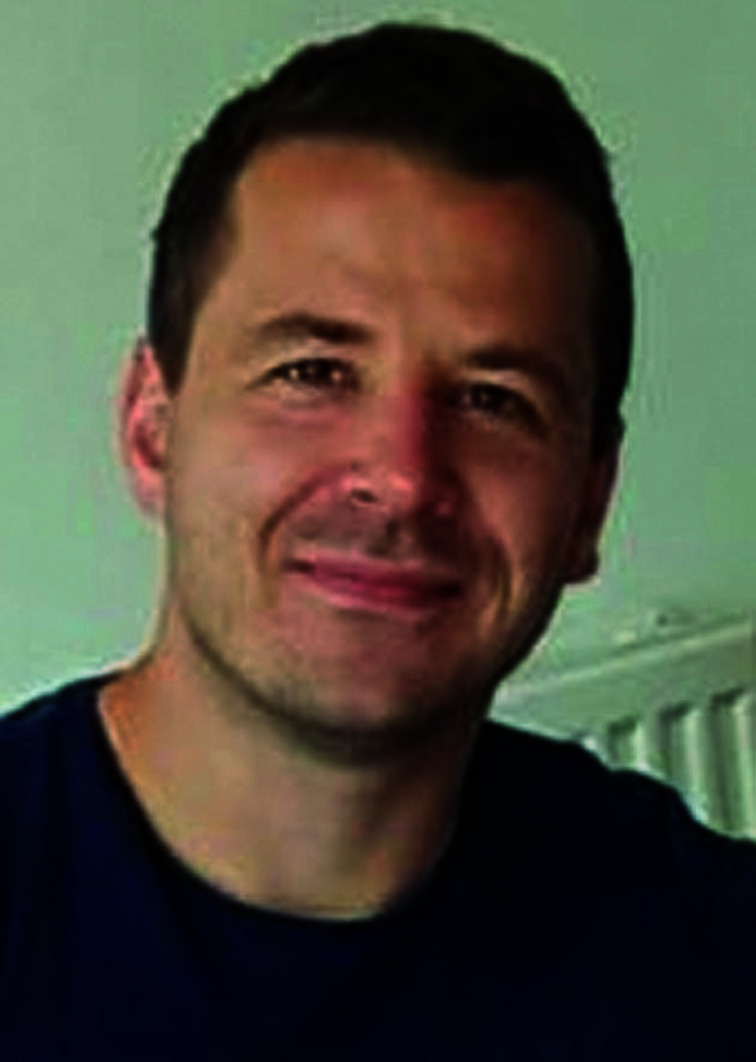

## Data Availability

Data sharing is not applicable to this article as no new data were created or analyzed in this study.
